# Habitat-related variation in daily activity patterns of Boutourlini’s blue monkey (*Cercopithecus mitis boutourlinii)* in Saja Forest, Kafa Biosphere Reserve, Southwest Ethiopia

**DOI:** 10.1186/s40850-026-00280-4

**Published:** 2026-07-28

**Authors:** Mulu Negesse, Tsegaye Gadisa, Tadesse Habtamu

**Affiliations:** 1 Department of Biology, College of Natural Sciences, Bonga University, Bonga, Ethiopia; 2 Department of Biology, College of Natural Sciences, Jimma University, Jimma, Ethiopia

**Keywords:** Activity budget, Biosphere reserve, *Cercopithecus mitis boutourlinii*, Habitat type, Ethiopia, Saja forest, seasonal variation

## Abstract

**Background:**

Activity budgets provide insights into how primates respond to habitat variation. Through behavioural flexibility, primates, including blue monkeys, can adjust their activity patterns and resource use under changing environmental conditions. This study compared the activity budgets of two Boutourlini’s blue monkey groups inhabiting different forest zones of Saja Forest: the core and transition zones. We collected Behavioral data from August 2024 to October 2025 using the instantaneous scan sampling method.

**Results:**

A total of 19,725 behavioral observations were recorded. Feeding was the dominant activity (29.6%), while times allocated for other activities were a smaller proportion (7.5%). The core-zone group spent significantly more time on feeding (33.0%), whereas those in the transition study group spent more time on movement (29.5%), and both showed statistically significant differences. Social activity was also higher in the transition zone group (13.5%), but it was not significant. Feeding was higher in the dry season (32.11%), whereas moving was higher in the wet season (28.67%). Diurnal patterns were observed, with feeding peaking in the morning and afternoon, while resting was highest at midday. Activity budgets varied among sex–age categories, with adult females showing the highest proportion of feeding activity (33.3%).

**Conclusions:**

These findings provide baseline information on the activity budgets of two Boutourlini’s blue monkey groups occupying different forest zones. The observed variation in activity budgets between study groups and across seasons suggests behavioural flexibility under different environmental conditions. Future studies involving multiple independent groups per habitat type and direct measurements of habitat characteristics are needed to better understand the ecological factors influencing behavioural variation and to support conservation planning.

**Supplementary Information:**

The online version contains supplementary material available at 10.1186/s40850-026-00280-4.

## Background

Understanding daily activity budgets is basic to behavioral ecology because they reflect how primates allocate time to essential behaviors such as feeding, resting, moving, and socializing [1, 2]. Activity budgets are shaped by multiple ecological factors, including food availability, habitat structure, predation risk, and social interactions [3–6]. Primates exhibit behavioral flexibility that enables them to adjust activity budgets to maximize energy intake while minimizing energetic costs under different environmental conditions [7–9]. Variation in activity budgets across habitat types and seasons therefore provides valuable insight into species’ ecological requirements and adaptive responses to environmental change [6].

Habitat type strongly influences primate behavior by determining the abundance, quality, and spatial distribution of food resources, as well as the availability of shelter and protection from predators [4, 7]. In relatively undisturbed forests, predictable resource availability and continuous canopy cover generally allow primates to exhibit consistent activity patterns [10]. In contrast, habitat degradation and fragmentation reduce food availability, disrupt canopy connectivity, alter microclimatic conditions, and increase human disturbance, often requiring individuals to spend more time travelling to find food and suitable resting sites [7, 11–13]. As a result, changes in activity budgets provide an important indicator of how primates respond to habitat alteration [9]. Habitat loss and fragmentation are major threats to tropical forests, reducing habitat quality, isolating forest patches, limiting movement, decreasing food availability, and altering microclimatic conditions and predation risk [7, 13].

In response to these environmental challenges, primates, including blue monkeys, often exhibit considerable behavioral flexibility, enabling them to adjust their activity patterns and resource use under changing ecological conditions [5, 14]. Understanding these adaptive responses is therefore essential for assessing how species cope with habitat modification [15]. In blue monkeys, daily activity patterns are closely associated with habitat characteristics, as the availability of food, water, and protective cover influences feeding, movement, and social behaviors such as grooming and breeding [16].

Boutourlini’s blue monkey (*Cercopithecus mitis boutourlinii*), an endemic Ethiopian subspecies classified as Vulnerable. This subspecies was first described from Shewa (parts of central Ethiopia) by the Russian explorer Augusto Boutourline [14]. Like other blue monkeys, this subspecies is highly arboreal and depends on intact forest habitats for foraging, movement, and refuge. Consequently, it is particularly susceptible to habitat degradation and fragmentation resulting from increasing human activities [17–19]. Such environmental changes are expected to influence its activity budgets by increasing travel costs, reducing resource availability, and altering the time allocated to feeding, moving, resting, and social behaviors [17, 19].

Despite its conservation importance, the behavioral ecology of Boutourlini’s blue monkey (*Cercopithecus mitis boutourlinii*) remains poorly understood. In the Kafa Biosphere Reserve, previous research has mainly focused on documenting the distribution of Boutourlini’s blue monkey [20], with no study examining activity budgets or comparing behavioural activity patterns between groups occupying different forest zones. Consequently, little is known about how this primate adjusts its behaviour under different habitat conditions, limiting our understanding of its ecological flexibility and habitat requirements.

To address this knowledge gap, the present study compared the activity budgets of two habituated Boutourlini’s blue monkey groups inhabiting different forest zones of the Saja Forest: one in the core zone and the other in the transition zone. We hypothesized that activity budgets would differ between the study groups and seasons. Specifically, we quantified and compared the time allocated to feeding, resting, moving, socializing, and other behaviors. However, since only one habituated group per habitat was studied and habitat characteristics such as food availability and vegetation structure were not measured, observed behavioral differences are interpreted as differences between study groups rather than as direct effects of habitat quality.

## Methods

### Description of the study area

**Fig. 1:**
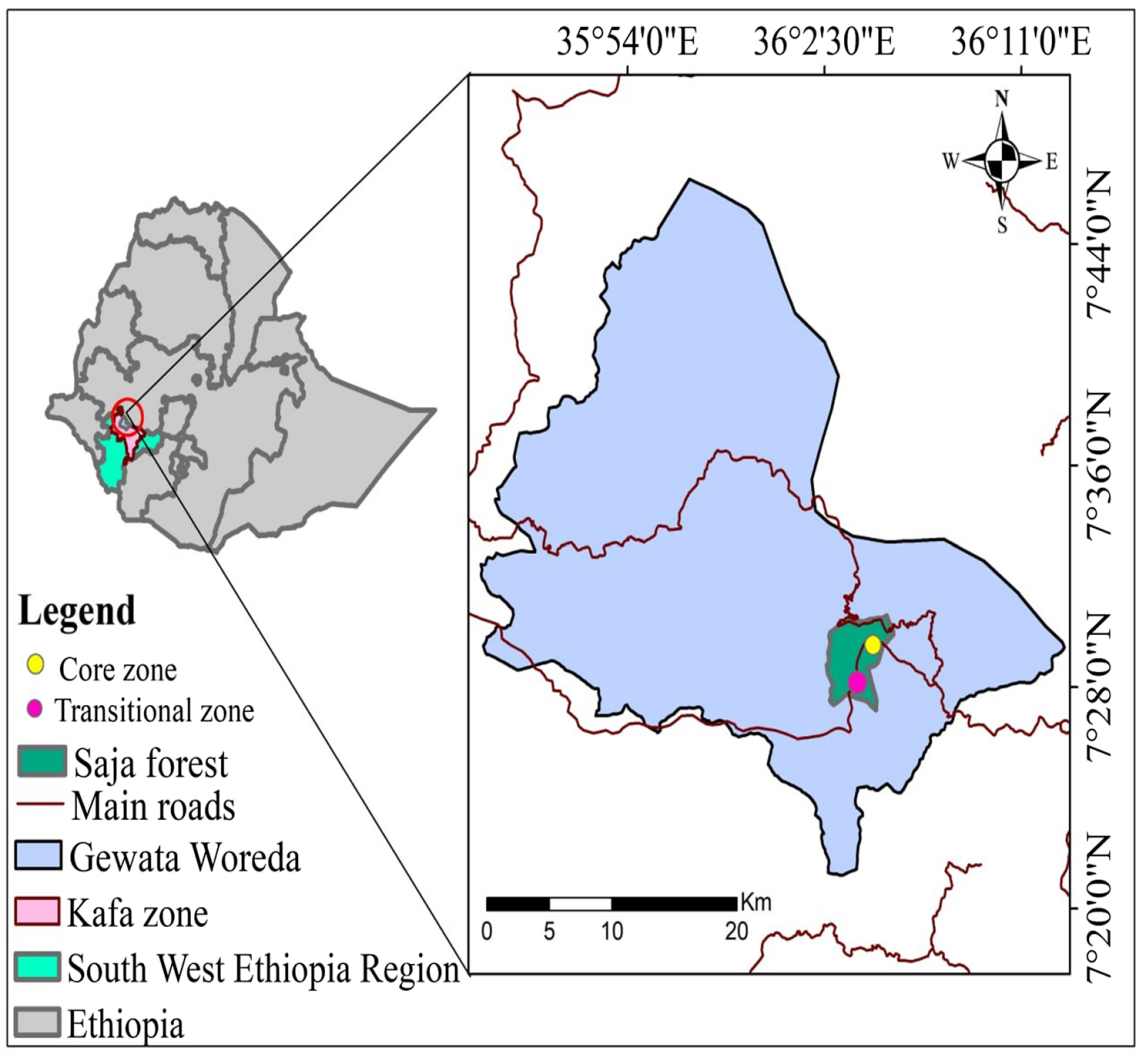
Map of the study area

The study was conducted in Saja Forest, Gewata District of the Kafa Biosphere Reserve(KBR), Southwest Ethiopia between (7°03′–7°05′ N, 35°50′–36°10′ E) (Fig. 1). The Kafa Biosphere Reserve is one of six UNESCO-recognized biosphere reserves in Ethiopia and is considered one of 34 global biodiversity hotspots due to its exceptional species richness [21]. Saja forest is located 547 km southwest of Addis Ababa and covers about 1,968 hectares [22]. The forest is one of the largest and most accessible in the KBR, and it is crossed by the main road that connects Bonga and Seyliem.

The forest is composed of core, buffer, and transition zones, which are influenced by surrounding agricultural activities and human settlements (Table 1). These diverse ecosystems make a great site for studying habitat-based variations in primate activity budgets. The area has bimodal rainfall, with distinct wet and dry seasons, mean annual temperatures ranging from 12.6 to 25.7 °C, and an annual rainfall of 2,235 mm.

Saja Forest supports five primate species, including the vulnerable Boutourlini’s blue monkey (*C. mitis. boutourlinii*), olive baboon (*Papioanubis*), guereza (*Colobus guerezaguereza*), grivet monkey (*Chlorocebus aethiops aethiops*), and de brazza’s monkey (*Cercopithecus neglectus*). Large mammals, such as African buffalo (*Syncerus caffer*), lion (*Pantheraleo*), and leopard (*Pantherapardus*), as well as over 250 bird species, are also present [21]. The surrounding area is used for subsistence farming, livestock rearing, and other forest products, including honey, shade coffee, and spices [22, 23]. Common crops include maize (*Zea mays*), barley (*Hordeumvulgare*), wheat (Triticumaestivum), Sorghum (*Sorghum bicolor)*, vegetables, fruits, coffee (*Coffe aarabica*), and enset (*Ensete ventricosum*).

**Table 1:** Description of habitat types in Kafa Biosphere Reserve

Habitat Type	Description
Core zone	The core zone is a strictly protected and undisturbed area designated for biodiversity conservation and research. It includes primary and riparian forests that support diverse species, including endemic and higher-order predators.
Transition zone	The transition zone is the outermost area, characterized by mixed natural and human-modified habitats such as farmland, grazing land, fragmented forest, plantation forest, and bamboo forest. It supports settlement and sustainable economic activities.

### Sampling design

A reconnaissance survey was conducted from June 2023 to April 2024 to identify the distribution of Boutourlini’s blue monkey, obtain baseline information on habitat characteristics, select suitable study sites, and select the study groups. Prior to behavioral observations, the researcher and a trained field assistant habituated two randomly selected study groups of Boutourlini’s blue monkey from May to July 2024 by following them at a distance that did not cause disturbance or flight responses. The study groups were identified based on distinctive natural markings, coat coloration, and group size [24]. When the monkeys passed through small openings in the forest, we took the opportunity to conduct group counts. Based on repeated counts, the total group size in the core zone group ranged from 37 to 41 individuals, while the group in the transition zone ranged from 21 to 23 individuals.

### Data collection

We conducted data collection from August 2024 to October 2025 for 15 months covering both the wet and dry seasons. Wet-season data were collected from August–October 2024 and June–October 2025, while dry-season data were collected from November 2024 to May 2025. Each study group was observed for 3–5 consecutive days per month. During wet season, field conditions were challenging due to heavy rainfall and poor visibility. As a result, data collection during this period was limited to three consecutive days per month to ensure observer safety and data quality while maintaining consistent sampling effort across groups.

Data collection was conducted daily from 06:30 to 17:30 h using instantaneous scan sampling at 15-minute intervals, with each scan lasting approximately 5 min [1, 5, 24, 25]. During each scan, the first activity observed for each visible individual was recorded. Individuals that were temporarily out of sight due to dense vegetation or movement were not recorded during that scan but were included in subsequent scans when they became visible. Activities were classified into feeding, moving, resting, socializing, and other behaviors (e.g., urination, defecation, vocalization, and drinking). Feeding was recorded when individuals were foraging, digging, and transferring to the mouth or chewing food items. Moving was recorded when Boutourlini’s blue monkeys were in locomotion such as walking, jumping, running, or climbing that resulted in changing of spatial position and not engaged in feeding or any form of social activity [25]. Resting covered inactive behaviors such as sitting or sleeping, and socialising included interactions such as grooming, play, aggression, and sexual behavior [26].

For each scan, the time, date, habitat type, activity, and age–sex class of each individual were recorded. Individuals were classified as adult male, adult female, sub-adult male, sub-adult female, and juvenile. Infants were excluded from scan sampling due to their dependence on mothers and limited independent activity ([24–26]. A total of five daily time intervals (H1–H5) were used to examine the diurnal activity pattern of the study group. To minimize sampling bias and avoid over-representation of conspicuous behaviours, group members were scanned systematically from left to right or right to left during each scan [5, 24], ensuring that each individual was recorded only once per scan.

### Statistical analysis

Behavioural data were analysed using IBM SPSS Statistics version 26, with statistical significance set at *p* < 0.05. To reduce temporal pseudoreplication arising from repeated scan sampling of the same study groups, behavioural observations were summarized into monthly values, which served as the statistical units for most of analyses. Activity budgets were calculated as the proportion of scan observations assigned to each behavioural category, expressed as a percentage of the total recorded observations. Monthly activity budgets were calculated using the same approach and were used as the statistical unit for descriptive summaries and non-parametric analyses. Because monthly percentage data were not normally distributed, the Mann–Whitney *U* test was used to compare activity budgets between the core-zone and transition-zone study groups and between the wet and dry seasons. Monthly variation in activity budgets was assessed using the Kruskal–Wallis test. Diurnal activity patterns across five daily time intervals (H1–H5) and differences among age–sex classes were analyzed using chi-square tests based on raw scan frequencies, with effect sizes quantified using Cramer’s *V*.

A two-way multivariate analysis of variance (MANOVA) was used to evaluate the effects of study group (core zone vs. transition zone), season (wet vs. dry), and their interaction on five behavioural variables (feeding, moving, resting, socializing, and other behaviours) using monthly behavioural counts. Counts were analyzed because they preserved the original multivariate structure of the observations, whereas percentages were reserved for descriptive and non-parametric analyses. Assumptions of normality, homogeneity of variances, and homogeneity of covariance matrices were assessed using the Shapiro–Wilk, Levene’s, and Box’s *M* tests, respectively. Because minor departures from normality were detected, multivariate effects were evaluated using Pillai’s Trace, which is robust to moderate violations of these assumptions.

The analysis was based on 15 monthly observations for each study group. Because only one habituated study group was observed in each habitat type, habitat was not independently replicated. Consequently, the analysis compares behavioural differences between the two study groups occupying different habitat conditions, and habitat-related effects should be interpreted with caution.

## Results

### Overall activity budget of Boutourlini’s blue monkeys

**Fig. 2:**
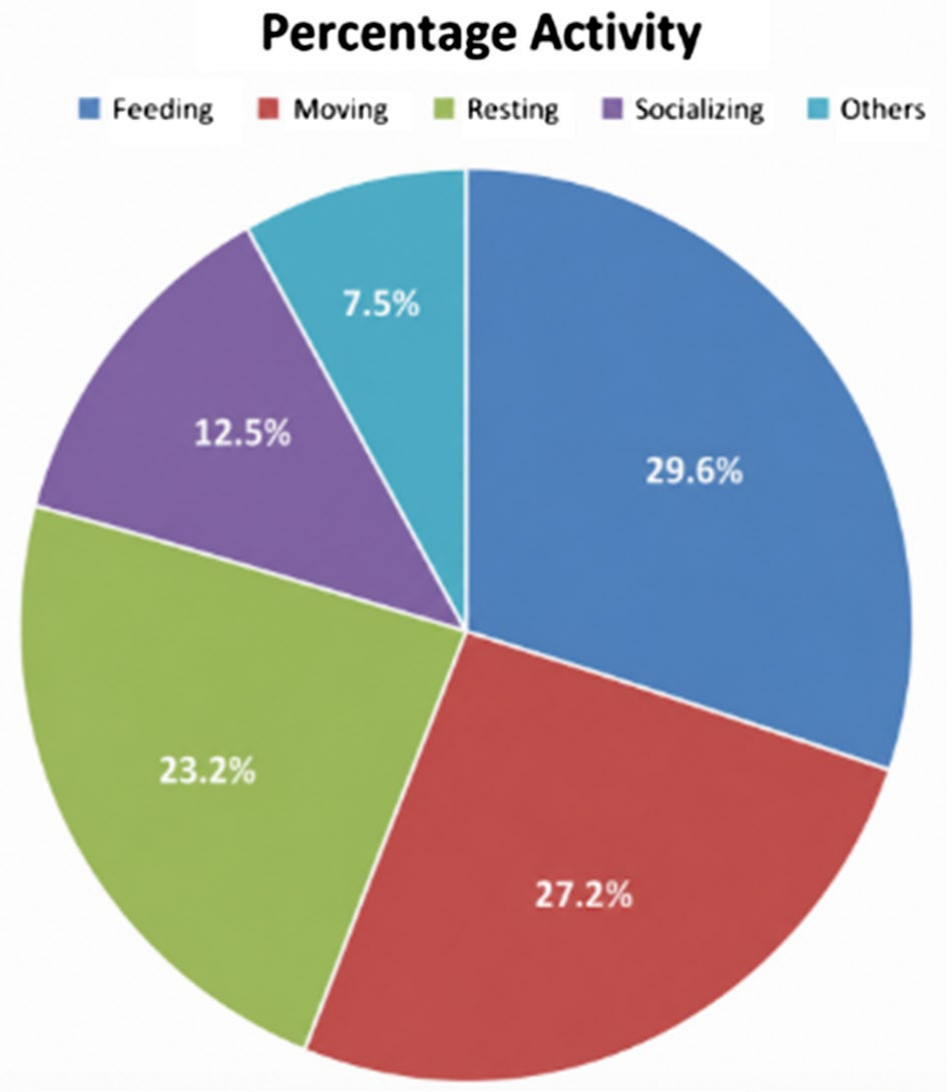
Overall activity budget (%) of Boutourlini’s blue monkeys during the study period

A total of 19,725 behavioral data were collected from 4,675 instantaneous group scans during 126 observation days, with 10,238 observations in the core zone group and 9,487 in the transition zone group. Feeding accounted for the largest proportion of the activity budget (29.6%), whereas other activities accounted for the smallest proportion (7.5%) (Fig. 2).

### Variation in activity budgets between core- and transition-zone study groups

**Fig. 3:**
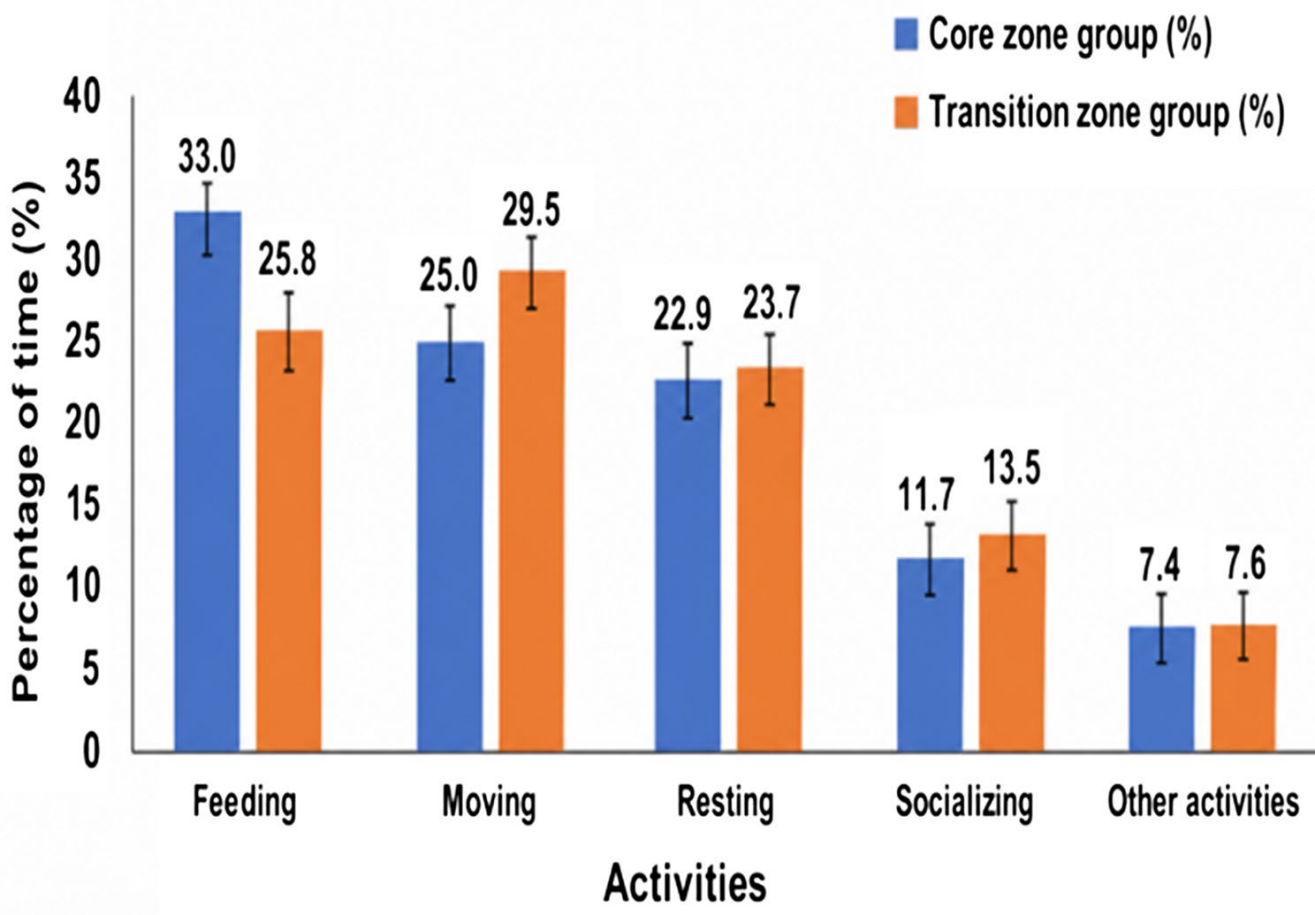
Habitat-based activity budget of Boutourlini’s blue monkey in Saja Forest

Activity allocation differed between the core-zone and transition-zone study groups (Fig. 3). The core-zone group spent significantly more time feeding than those in the transition-zone group (U = 22.0, *p* < 0.001). Moving was significantly higher in the transition-zone group (U = 39.0, *p* = 0.002), whereas resting did not differ significantly between the two study groups (U = 98.0, *p* = 0.548). Socializing did not differ significantly between the two study groups (U = 70.0, *p* = 0.078). Other activities were also similar between the study groups, with no significant difference (U = 87.5, *p* = 0.300).

### Seasonal variation in activity budgets of study groups

Seasonal comparisons of monthly activity budgets showed that feeding and moving activities differed significantly between the wet and dry seasons (Table 2). Feeding was higher in the dry season (32.11%) than in the wet season (27.17%). Moving was higher in the wet season (28.67%) than in the dry season (25.84%). Resting was slightly higher in the wet season (23.54%) than in the dry season (22.84%), with no significant difference. Socializing and other activities were slightly higher in wet season. We applied Mann–Whitney U tests to compare seasonal differences in activity budgets.

**Table 2:** Seasonal differences in activity time budgets of Boutourlini’s blue monkeys based on Mann–Whitney U tests

Activity	Season	Mean rank	Mann–Whitney U	Z	*p*-value
Feeding	Wet	4.75			
	Dry	11.71	2.0	−3.01	**0.003**
Moving	Wet	10.31			
	Dry	5.36	9.5	−2.14	**0.032**
Resting	Wet	9.00			
	Dry	6.86	20.0	−0.93	0.355
Socializing	Wet	9.25			
	Dry	6.57	18.0	−1.16	0.247
Others	Wet	8.63			
	Dry	7.29	23.0	−0.58	0.563

Values are based on monthly activity

## Monthly variation in activity budgets

**Fig. 4:**
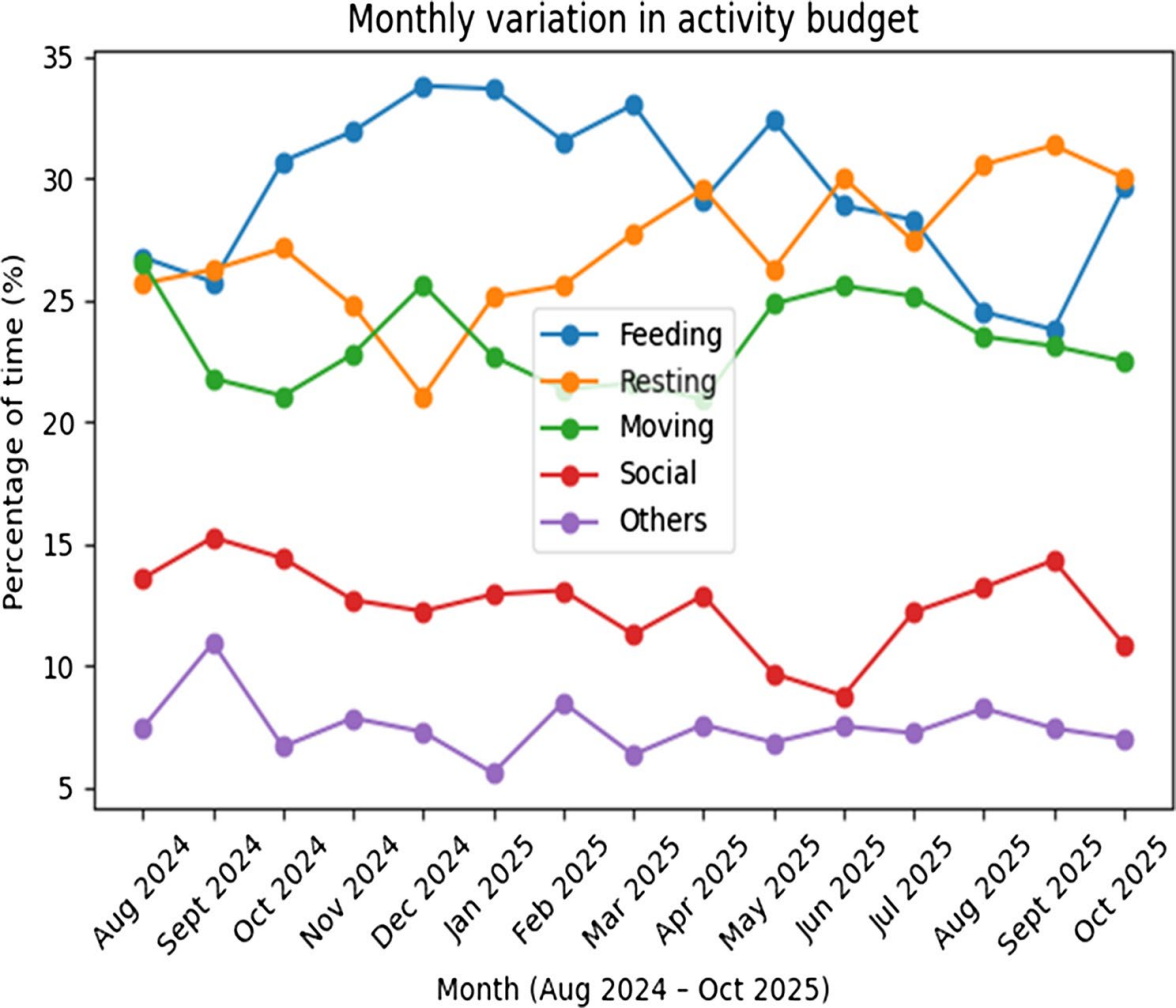
Monthly variation in activity budgets of *Cercopithecus mitis boutourlinii*

Monthly activity budgets showed temporal variation (Fig. 4), with feeding consistently representing the dominant activity throughout the study period. Resting and moving showed moderate fluctuations, while socializing and other activities remained relatively low across months. However, Kruskal–Wallis tests indicated that these monthly differences were not statistically significant for any activity category: feeding (H = 10.72, df = 14, *p* = 0.708), resting (H = 13.46, df = 14, *p* = 0.491), moving (H = 8.43, df = 14, *p* = 0.866), socializing (H = 13.70, df = 14, *p* = 0.472), or other activities (H = 10.35, df = 14, *p* = 0.736).

### Effects of study group and season on activity budgets

Activity budgets of Boutourlini’s blue monkeys varied between study groups and seasons (Table 3). Feeding was the most frequent activity in both the core and transition study groups, followed by moving and resting. The core zone group showed higher feeding activity (30.26–36.50%) compared to the transition zone group (24.07–27.71%). In contrast, the transition group spent more time moving (28.06–30.83%) than the core group (23.62–26.50%). Resting, socializing, and other behaviors showed relatively similar values between study groups and across seasons, with only minor variation.

Two-way MANOVA revealed significant multivariate effects of study group on activity budgets (Pillai’s Trace = 0.746, F(5, 22) = 12.939, *p* < 0.001) and season (Pillai’s Trace = 0.538, F(5, 22) **=** 5.118, *p* = 0.003). However, the interaction between study group and season was not significant (Pillai’s Trace = 0.117, F(5, 22) = 0.584, *p* = 0.712).

**Table 3:** Activity budgets (mean ± SD %) of Boutourlini’s blue monkeys across study groups and seasons

Activity	Study group	Wet season (Mean ± SD)	Dry season (Mean ± SD)	Overall (Mean ± SD)
Feeding	Core group	30.26 ± 3.72	36.50 ± 2.55	33.17 ± 4.48
	Transition group	24.07 ± 2.20	27.71 ± 3.29	25.77 ± 3.25
Moving	Core group	23.62 ± 3.54	26.50 ± 3.07	25.16 ± 3.51
	Transition group	28.06 ± 2.33	30.83 ± 3.03	29.54 ± 2.99
Resting	Core group	23.45 ± 2.30	22.12 ± 3.80	22.83 ± 3.05
	Transition group	23.62 ± 4.34	23.55 ± 2.13	23.59 ± 3.37
Socializing	Core group	11.84 ± 2.86	11.15 ± 2.54	11.52 ± 2.64
	Transition group	13.86 ± 2.41	13.03 ± 1.89	13.47 ± 2.15
Others	Core group	7.95 ± 2.31	6.60 ± 1.26	7.32 ± 1.96
	Transition group	7.61 ± 1.42	7.66 ± 1.70	7.63 ± 1.50

Values are mean ± standard deviation (SD). Percentages show time allocation to each activity. Two-way MANOVA showed significant effects of study group and season, but no significant interaction between them. Therefore, results for habitat–season combinations are presented only as descriptive statistics

### Activity allocation by time interval

Activity budgets showed clear daily variation (Table 4). Feeding peaked in the early morning (H1) and afternoon (H5), while resting was highest at mid-day (H3). In contrast, moving activity remained relatively consistent throughout the day. Socializing showed moderate variation, while other activities remained low and showed minor variation. A chi-square test indicated a significant association between activity and time interval (χ² = 27,872, df = 16, *p* < 0.001).

**Table 4:** Mean (± SD) % of time allocated to different activities across daily time intervals

Activity	H_1_ (%)	H_2_ (%)	H_3_ (%)	H_4_ (%)	H_5_ (%)	Mean ± SD (%)
Feeding	41.96	26.35	19.15	27.22	31.75	**29.29 ± 8.40**
Moving	31.03	32.87	17.96	27.14	26.52	**27.10 ± 5.76**
Resting	11.99	21.81	37.42	26.13	20.22	**23.51 ± 9.31**
Socializing	8.59	11.14	17.42	11.58	14.12	**12.57 ± 3.35**
Others	6.44	7.83	8.02	7.91	7.38	**7.52 ± 0.65**

H1 = 06:30–08:30 h; H2 = 08:45–10:45 h; H3 = 11:00–13:00 h; H4 = 13:15–15:15 h; H5 = 15:30–17:30 h

### Activity budgets across sex–age categories

Activity budget allocation differed significantly among sex–age categories (Pearson’s χ² = 356.81, df = 16, *p* < 0.001; Table 5). Adult females devoted the greatest proportion of time to feeding (33.3%), whereas juveniles allocated comparatively more time to socializing (19.3%). Sub-adult males and females exhibited higher proportions of movement activity (28.2% and 27.9%, respectively) than adult males (24.8%). Resting activity was most pronounced in adult males (27.1%) and least in juveniles (17.9%). Other activities consistently represented a small proportion of the activity budget across all sex–age categories. Although the association between activity budget and sex–age category was statistically significant, the effect size was very small (Cramer’s V = 0.08), indicating that the differences in activity budgets among sex–age categories were weak.

**Table 5:** Percentage of time spent on different activities by sex–age categories

Activity	AM (%)	AF (%)	SAM (%)	SAF (%)	JUV (%)
Feeding	29.5	33.3	26.3	27.9	29.1
Moving	24.8	27.9	28.2	27.9	26.7
Resting	27.1	22.6	24	24.2	17.9
Socializing	8.6	10	14.2	12.7	19.3
Others	10	6.3	7.3	7.3	7

## Discussion

### Overall activity budget

The activity budget of Boutourlini’s blue monkeys in the study area was dominated by feeding, followed by moving and resting, while socializing and other activities accounted for smaller proportions. This general pattern is consistent with many diurnal cercopithecoidae primates, where feeding and locomotion typically represent the largest components of daily time allocation. Similar findings have been reported in different parts of Ethiopia, including studies conducted in the Odobullu Forest [23], on geladas in Debre Berhan [26], Wollo [27], the Kotu Forest in northern Ethiopia [28], Jibata Forest [19] and around Debre Libanos [29].

The high proportion of feeding activity reflects the central role of foraging in daily energy intake. Variation in feeding and movement suggests flexible adjustment of time allocation to obtain sufficient food resources under changing conditions. Increased movement may reflects travel between feeding locations, while resting may represent periods of reduced activity following intensive foraging and locomotion. Lower investment in social and other activities suggests that these behaviors are maintained at relatively stable but reduced levels compared to essential maintenance behaviors. In general, the predominance of feeding in the activity budget suggests that foraging represents the principal behavioral priority of Boutourlini’s blue monkeys, with other activities adjusted around this essential requirement.

### Habitat-related variation in activity budgets

The core-zone group spent significantly more time feeding, whereas the transition-zone group spent more time moving; both behaviours differed significantly between the study groups. These results indicate variation in behavioral time allocation between the two study groups. The higher feeding activity in the core study group may reflect differences in how individuals used available resources within their home ranges, while the greater movement in the transition study group may indicate differences in ranging patterns or spatial use. However, these explanations remain hypothetical because detailed data on habitat characteristics and resource distribution were not collected. Although, the difference in group sizes between the two study groups may have influenced activity budgets. Resting, socializing, and other activities were slightly more frequent in the transition-zone group than in the core-zone group; however, these differences were not statistically significant.

Comparable patterns have been reported in other primates inhabiting disturbed or heterogeneous environments. Other cercopithecines show variation in feeding and movement patterns across different habitat conditions [17, 24], while increased movement and social adjustments have been documented in primate populations living in fragmented forests [13, 30, 31]. Although the ecological drivers of these patterns may differ among studies, they suggest that activity allocation can vary across groups in response to differences in environmental context. It should be noted that these interpretations are tentative because habitat characteristics such as vegetation structure, canopy cover, and resource availability were not directly measured. In addition, because only one study group was observed per habitat type, group-specific variation cannot be separated from habitat effects. Therefore, the observed differences should be interpreted as comparisons between two study groups rather than general habitat-level patterns.

### Seasonal variation in activity budgets

Season influenced feeding and movement activity budgets, whereas resting, socializing, and other activities remained relatively stable. Feeding activity was significantly higher during the dry season, suggesting that individuals allocated more time to foraging during this period. Similar seasonal increases in feeding have been reported for other primate species, where changes in environmental conditions are associated with adjustments in foraging behavior [24, 32, 33]. However, because food availability was not directly measured in this study, the underlying factors responsible for this seasonal difference cannot be determined.

In contrast, moving activity was significantly higher during the wet season. This finding differs from studies reporting greater movement during periods of reduced food availability [17, 34–36], indicating that seasonal patterns of locomotion may vary among populations and habitats. The higher movement observed in the wet season may reflect seasonal changes in ranging or resource use, although these factors were not evaluated in the present study.

Resting, socializing, and other activities did not differ significantly between seasons, indicating that these components of the activity budget were relatively stable throughout the study period. This stability suggests that seasonal variation primarily affected feeding and movement, while the allocation of time to other behaviors remained largely unchanged, consistent with observations from other primate studies [24, 32–37]. Collectively, seasonal variation influenced feeding and movement more than other behavioral categories, indicating that these activities were the most responsive to seasonal changes during the study period.

### Monthly variation in activity budgets

Although monthly fluctuations in activity budgets were observed, none of the behavioral categories differed significantly among months. Feeding consistently remained the dominant activity, whereas resting and moving showed only modest fluctuations. This stability suggests that Boutourlini’s blue monkeys maintained a consistent pattern of time allocation throughout the study period despite short-term temporal variation.

The limited monthly variation may indicate that individuals adjusted the proportion of time devoted to particular activities without altering their overall behavioral strategy. Such behavioral consistency may be advantageous in environments where conditions change gradually rather than abruptly, allowing animals to maintain essential activities while making only minor adjustments in daily time allocation. However, because environmental variables such as food availability and vegetation characteristics were not quantified, the specific drivers of these monthly fluctuations cannot be determined from the present study.

Similar patterns have been reported in other primates, where modest temporal fluctuations in feeding and resting occur without significant changes in overall activity budgets. For example, Gelada exhibit temporal variation in activity without significant differences in overall activity allocation [33], while Vervet monkey show flexible adjustments in feeding and resting while maintaining relatively stable activity budgets [38, 39]. The consistency of these findings with the present study suggests that monthly fluctuations in specific behaviors do not necessarily alter the overall pattern of activity allocation in Boutourlini’s blue monkeys.

### Effects of study group and season on activity budgets

Activity budgets differed significantly between the two study groups and across seasons, whereas the absence of a significant study group × season interaction indicates that seasonal changes in activity allocation were broadly similar in both groups. This suggests that differences between the study groups were maintained throughout the study period rather than varying with season. The higher feeding activity observed in the core study group and the relatively greater movement and socializing in the transition zone study group indicate differences in behavioral time allocation between the two groups. These differences may reflect variation in local environmental conditions, difference in group sizes between the two study groups, patterns of space use, or other group-specific characteristics that influenced how individuals allocated time among activities. However, because only one habituated study group was observed in each habitat and habitat characteristics (e.g., vegetation structure, canopy cover, and resource availability) were not directly measured, the underlying causes of these differences cannot be determined from the present study.

Season also influenced activity budgets, primarily through changes in feeding and movement, whereas the lack of a significant interaction indicates that both study groups’ responded to seasonal variation in a comparable manner. This consistency suggests that seasonal adjustments in behavior occurred irrespective of the differences observed between the two study groups.

Similar patterns have been reported in other primates. Increased feeding during the dry season has been documented in Susgen Natural Forest [33], whereas differences in activity budgets among populations occupying different environments have been reported from around Debre Libanos [29]. Seasonal variation in activity allocation has also been described for Gelada near Abogedam Church [26] and Blue monkey [40]. Although the factors underlying these patterns may differ among studies, the present findings are consistent with evidence that primate activity budgets vary across groups and seasons while maintaining broadly similar seasonal responses. In summary, the findings suggest that both study group and season influenced activity allocation in Boutourlini’s blue monkeys, whereas the absence of a study group × season interaction indicates that seasonal patterns were comparable between the two study groups.

### Diurnal variation in activity budgets

The activity budgets of Boutourlini’s blue monkeys varied across the day, with feeding being most frequent during the early morning and late afternoon, whereas resting peaked around mid-day. In contrast, movement, socializing, and other activities showed comparatively little variation throughout the day. These patterns indicate that the allocation of time to feeding and resting changed over the course of the day, while other behavioral categories remained relatively stable. The observed diurnal variation may reflect adjustments in behavioral time allocation in response to changing conditions during the day. The concentration of feeding during the morning and late afternoon, together with increased resting around mid-day, is consistent with diurnal patterns reported for other primates. However, because environmental variables such as food availability were not measured, the factors underlying these daily changes cannot be determined from the present study.

Comparable patterns have been reported in several primate species, where feeding is concentrated during the morning and late afternoon and resting increases around mid-day [6, 17] and [41–43]. Although the mechanisms responsible for these patterns may differ among species and study sites, the present findings are consistent with evidence that primates adjust the timing of specific activities while maintaining a broadly similar daily activity budget. The present findings suggest that Boutourlini’s blue monkeys exhibited predictable diurnal variation in feeding and resting, whereas movement, socializing, and other activities remained relatively stable throughout the day.

### Sex–age differences in activity budgets

Activity allocation differed significantly among sex–age categories, with adult females allocating more time to feeding, juveniles showing higher levels of social activity, and adult males spending more time resting. These differences indicate that behavioral time allocation varied among age and sex classes during the study period. The observed variation may reflect differences in the behavioral roles or energetic requirements of the different sex–age categories. However, because reproductive status, dominance relationships, and other social or ecological factors were not evaluated, the mechanisms underlying these differences cannot be determined from the present study.

Comparable patterns have been reported in other primates. Juveniles generally engage more frequently in social activities than adults [44], and some studies shown that feeding dominates activity budgets across sex–age classes, with variation in social behavior [33, 34 ]. Greater resting by adult males has also been documented in other cercopithecine primates [6, 34, 45]. Although the underlying causes may differ among species, these studies indicate that differences in activity allocation among sex–age categories are common in primates. Taken together, the present findings indicate that sex–age differences contributed to variation in activity budgets; however, the relatively small effect sizes suggest that these differences accounted for only a modest proportion of the overall behavioral variation, with additional ecological and social factors likely also influencing activity allocation [46, 47].

### Study limitations

A key limitation of this study is that comparisons were based on only one habituated group per habitat type; therefore, habitat effects cannot be fully separated from group-specific behavioural characteristics. Although monthly aggregation of scan samples reduced temporal pseudoreplication, all observations were derived from only two groups. Thus, the results should be interpreted as comparisons between study groups rather than fully replicated habitat-level effects. Future studies incorporating multiple independent groups per habitat type and direct measurements of habitat characteristics would strengthen inferences about environmental influences on activity budgets.

## Conclusion and recommendation

This study documented differences in activity budgets between two Boutourlini’s blue monkey groups occupying the core and transition zones. The core-zone group devoted more time to feeding, whereas the transition-zone group spent relatively more time moving and socializing. Seasonal, diurnal, and sex–age variations further indicate behavioural adjustments under different environmental conditions. These findings provide baseline information on the activity patterns of Boutourlini’s blue monkeys and highlight the need for further research on factors influencing behavioural variation. Future studies should include multiple independent groups per habitat type, groups with comparable sizes, and direct measurements of habitat characteristics, food availability, and nutritional quality to better distinguish habitat effects from group-specific influences.

## Supplementary Information

Below is the link to the electronic supplementary material.

Supplementary Material 1

## Data Availability

The datasets used in this study are available upon reasonable request from the corresponding author.
