# Supplementary material for: Habitat-related variation in daily activity patterns of Boutourlini’s blue monkey (Cercopithecus mitis boutourlinii) in Saja Forest, Kafa Biosphere Reserve, Southwest Ethiopia
Source: BMC Zool. 2026 Jul 28;11:24. doi: 10.1186/s40850-026-00280-4 (PMC13419307; doi:10.1186/s40850-026-00280-4)
Supplement: Supplementary file 1 — Supplementary Material 1 [file 40850_2026_280_MOESM1_ESM.docx]

|  |  |  |  |  |  |  |  |  |  |  |  |  |  |  |
| --- | --- | --- | --- | --- | --- | --- | --- | --- | --- | --- | --- | --- | --- | --- |

**Appendix 2**. Data sheet used for behavioural activity and event patterns (daily cycle)

Key Am=Adult male Sam= Sub adult male, Jm= Juvenile male, Af= Adult female Saf= Sub adult female, Jf= Juvenile female, U = Unknown)

| Time of the day | Habitat  type | GPS  Point | Sex/age categories | | | |  | | | | | | | | | Behavioural activities | | | |  | |  | | | |  |  |  |
| --- | --- | --- | --- | --- | --- | --- | --- | --- | --- | --- | --- | --- | --- | --- | --- | --- | --- | --- | --- | --- | --- | --- | --- | --- | --- | --- | --- | --- |
|  |  |  |  |  |  |  |  | | | | | | | | | Feeding | | | Moving | Resting | Socializing | | Others | |  |  |  |  |
|  |  |  | Am | Sam | Jm | | | Af | | Saf | | Jf | | U | |  | |  | |  |  | |  | |  | |  |  |
|  |  |  |  |  |  | | |  | |  | |  | |  | |  | |  | |  |  | |  | |  | |  |  |
|  |  |  |  |  |  | | |  | |  | |  | |  | |  | |  | |  |  | |  | |  | |  |  |
|  |  |  |  |  |  | | |  | |  | |  | |  | |  | |  | |  |  | |  | |  | |  |  |
|  |  |  |  |  |  | | |  | |  | |  | |  | |  | |  | |  |  | |  | |  | |  |  |
|  |  |  |  |  |  | | |  | |  | |  | |  | |  | |  | |  |  | |  | |  | |  |  |
|  |  |  |  |  |  | | |  | |  | |  | |  | |  | |  | |  |  | |  | |  | |  |  |
|  |  |  |  |  | |  | | |  | |  | |  | |  | |  |  | |  |  | |  |  | | | |  |
|  |  |  |  |  | |  | | |  | |  | |  | |  | |  |  | |  |  | |  | | | |  |  |
|  |  |  |  |  | |  | | |  | |  | |  | |  | |  |  | |  |  | |  | | | |  |  |
|  |  |  |  |  | |  | | |  | |  | |  | |  | |  |  | |  |  | |  | | | |  |  |
